# Versatile Layer-By-Layer Highly Stable Multilayer Films: Study of the Loading and Release of FITC-Labeled Short Peptide in the Drug Delivery Field

**DOI:** 10.3390/ma12081206

**Published:** 2019-04-12

**Authors:** Kun Nie, Xiang Yu, Navnita Kumar, Yihe Zhang

**Affiliations:** 1 Beijing Key Laboratory of Materials Utilization of Nonmetallic Minerals and Solid Wastes, National Laboratory of Mineral Materials, School of Materials Science and Technology, China University of Geosciences (Beijing), Beijing 100083, China; nk@cugb.edu.cn; 2 Department of Chemistry and Biochemistry, University of California, Los Angeles, CA 90095, USA; navnitakumar77@gmail.com

**Keywords:** mesoporous silica, layer-by-layer, FITC-peptide, hyaluronic acid, multilayer film, host-guest interaction

## Abstract

A viable short FITC-peptide immobilization is the most essential step in the fabrication of multilayer films based on FITC-peptide. These functional multilayer films have potential applications in drug delivery, medical therapy, and so forth. These FITC-peptides films needed to be handled with a lot of care and precision due to their sensitive nature. In this study, a general immobilization method is reported for the purpose of stabilizing various kinds of peptides at the interfacial regions. Utilizing Mesoporous silica nanoparticles can help in the preservation of these FITC-peptides by embedding themselves into these covalently cross-linked multilayers. This basic outlook of the multilayer films is potent enough and could be reused as a positive substrate. The spatio-temporal retention property of peptides can be modulated by varying the number of capping layers. The release speed of guest molecules such as tyrosine within FITC-peptide or/and adamantane (Ad)-in short peptides could also be fine-tuned by the specific arrangements of the multilayers of mesoporous silica nanoparticles (MSNs) and hyaluronic acid- cyclodextrin (HA-CD) multilayer films.

## 1. Introduction

Layer-by-layer self-assembly technology is a useful method to prepare multilayer films with a thickness which can be controlled even down to the nanoscale [1,2,3,4,5], and is among one of the most widely used techniques to weave the organic-inorganic multilayer films together [6,7,8,9,10]. Mesoporous silica nanoparticles (MSNs) have been widely used in adsorption and drug delivery due to their unique pore structure, large specific surface area and good biocompatibility [11]. Some of these can directly act as catalysts as well because the ordered mesoporous material can accelerate the diffusion rate of the product. The selectivity is 100% and the conversion rate is up to 90%. Due to the flexibility of the structure and the narrow pore distribution, the doping of metal oxide and other complexes to these ordered mesoporous material matrix makes them better catalysts. Biological macromolecules, such as enzymes, proteins, nucleic acids, etc., have molecular sizes less than 10 nm, while viruses have sizes around 30 nm. However, the ordered mesoporous materials have quite a range of pore sizes between 2 and 50 nm. Due to the non-toxicity of these MSNs, they play an important role in the decomposition and fixation of enzymes, proteins and other substances. The mesoporous silica used for a particular function needs to be of a particle size, otherwise it will have an adverse effect [11,12,13]. A lot of research has been done in the area of multilayer films conjoining polymer and inorganic nanoparticles [14,15,16], and this can be attributed to the variable functionalities from the organic and inorganic parts of multilayer films [17,18,19,20]. The multilayer films which incorporate different inorganic parts can generally be prepared either by forming a covalent cross-link or by utilizing the non-covalent interactions between the polymer and the inorganic parts [21,22,23,24,25]. According to the literature, under ultraviolet irradiation, poly (allylamine hydrochloride) (PAH) and 4,4′-diazostilbene-2,2’-disulfonic acid disodium salt (DAS) can form covalent cross-linked layers in situ [26]. Non-covalent multilayer films have been obtained by using different kinds of supramolecular interactions [27,28,29], electrostatic interactions [30], hydrophobic interactions [29], host–guest interactions [30,31,32] and so on and so forth.

The knowledge of diffusion mechanism of fluorescent agent in these multilayers is very important and can provide a deeper perspective into the molecular interaction in organisms, soft material systems, and various advanced functional films [33,34,35]. Due to their good absorptivity, water solubility, and fluorescence quantum yield, Fluorescein derivatives, especially Fluorescein Isothiocyanate (FITC) have become the most popular fluorescent reagents in this field. Owing to their wide fluorescence emission range, their good performance on photo-bleaching and fluorescence quenching on conjugation to biopolymers, the scope of the application can be widened for FITC-based dyes and their conjugates [36].

Hyaluronic acid (HA) consists of repeatable disaccharides of the β-glucuronic acid and N-acetyl-d-glucosamine [37]. Hyaluronic gel is found in synovial fluid and is the main component of a glycosaminoglycan superstructure complex which is associated with different polysaccharides such as the chondroitin sulfate [38]. Hyaluronic acid can anchor to the surface of the cell by attaching itself to the cell surface receptors. HA is an upcoming drug delivery molecule for soft tissue repair and regeneration [39]. Studies to control the host-guest interaction within supramolecular structures are gradually rising because of their potential application as stimuli-responsive hydrogels, nanoparticles, smart biosensor devices and so on [40,41].

Amino-betacyclodextrin (CD) is a member of cyclic oligosaccharides and consists of the lipophilic central cavity and a hydrophilic outer surface [42,43]. Amino-betacyclodextrin has a large structure with a many hydrogen donors and acceptor groups. However, these groups are impermeable to the lipophilic films, making them difficult to use as a drug delivery vehicle. To enhance aqueous solubility of those poorly soluble drugs and to also raise their bioavailability, cyclodextrin has been utilized as a complexing agent [44]. CD can be grafted onto HA by chemical synthesis, and then the properties of both the moieties in the complex can be utilized. The grafting rate can be measured by ^1^H NMR [45]. In addition, there is supramolecular hydrophobic host-guest interaction between adamantine (Ad) and cyclodextrin (CD) [46]. Peptides with Ad can be used as targets to study the effect of host-guest interactions in molecular diffusion. At present, there are many reported articles on the host-guest inclusion phenomena that delays the release of peptides [47].

In this paper, we have explored a new method to construct multilayer film materials that effectively preserves different kinds of short peptides. Mesoporous silica serves as a short peptide-container and functional multilayers were embedded in these via electrostatic interactions. Because of the exquisite film structure, the magnitude of the supramolecular interactions within the multilayer films can be modulated. The release curves of the short peptides vary with the number of laminated multilayers. The supramolecular force-delayed molecular release was taken as an example to further demonstrate the functions of the reported peptides immobilization strategy. This technique can be utilized to prepare multilayers which can further be used to control the fabrication of these versatile multilayer films.

## 2. Materials and Methods

### 2.1. Materials and Instruments

The following chemicals were used without any further treatment: Sodium hyaluronate (HA, 95%), amino-betacyclodextrin, fluorescein isothiocyanate (FITC, purity > 90), phosphate buffer solution (PBS) and peptides (FITC-RGD & FITC-RGD-Ad) modified with fluorescein isothiocyanate were provided by Sinopharm Chemical Reagent Beijing Co., Ltd (Beijing, China). Mono-(6-Amino-6-deoxy)-Beta-cyclodextrin was bought from Shandong Binzhou Zhiyuan Biotechnology Co., Ltd (Shandong, China). Peptides (FITC-(SGGYGGS)_4_ & FITC-(SGGSGGS)_4_) were bought from LifeTein LLC (Beijing, China). Cetyltrimethylammonium bromide surfactant (CH_3_(CH_2_)_15_N(CH_3_)_3_Br, CTAB), tetraethoxysilane (TEOS) and Poly(allylamine hydrochloride) (PAH, Mw = 15,000) were purchased from Sigma-Aldrich (St. Louis, MO, USA). 4,4′-diazostilbene-2,2′-disulfonic acid disodium salt (DAS), N-Hydroxy succinimide (NHS) and 1-(3-Dimethylaminopropyl)-3-ethylcarbodiimide hydrochloride (EDC) were purchased from TCI (Tokyo Chemical Industry, Tokyo, Japan). Only deionized water was used for all the syntheses.

UV-vis spectra were obtained using a Hitachi U-3900 spectrophotometer (Hitachi, Tokyo, Japan). The Surface morphologies of multilayer films were characterized using transmission electron microscope (TEM) (TEM, Tecnai T12, Field Electron and Ion Company, FEI, Hillsboro, OR, USA). TEM experiments were carried out on a Titan S/TEM (Field Electron and Ion Company, FEI, Hillsboro, OR, USA) microscope. ^1^H NMR spectra were obtained on Bruker Avance III 400MHz WB (Bruker, Baden, Switzerland). The Brunauer-Emmett-Teller(BET)-Barret-Joyner-Halenda(BJH) BET-BJH data were obtained from the Autosorb-iQ2 (Quantachrome, Boynton Beach, FL, USA).

### 2.2. The Preparation of HA-β-CD Gels

HA-CD gel was prepared according to literature [48]. In a 100 mL Erlenmeyer flask, 0.3 g of sodium hyaluronate and 50 mL of morpholinoethanesulfonic buffer (MES) were added and rapid stirred for 6 h. Subsequently, 0.285 g of NHS and 0.342 g of EDC were added into the Erlenmeyer flask. Then the mixture was stirred for another hour. To the mixture, 0.1686 g of amino cyclodextrin was added, followed by stirring for 24 h. The detailed experimental procedure and the sample codes are listed in Table S1. ^1^H NMR spectroscopy was used to determine the degree of grafting of HA-CD. The graft ratio was calculated by using the integral area of the ^1^H NMR spectrum. As shown in Figure S1, the peak at 5.10 ppm represents the proton of amino (NH) (5.10 ppm) group and its integral area was 0.11. Therefore, the integral area of each hydrogen atom of the aminocyclodextrin was 0.11/7. Similarly, the peak at 2.05 ppm represents the proton of CH_3_ (2.05 ppm) group and its integral area was 1.00. Therefore, the integral area of each hydrogen atom of hyaluronic acid was 1/3. Grafting ratio was obtained by dividing the integral area of each hydrogen atom of amino cyclodextrin by the integral area of each hydrogen atom of hyaluronic acid. So, according to the ^1^H NMR (400 MHz, D_2_O) data, the grafting rate of HA-CD was 4.71%.

### 2.3. The Synthesis of Mesoporous Silica Nanoparticles (MSNs)

**Figure 1:**
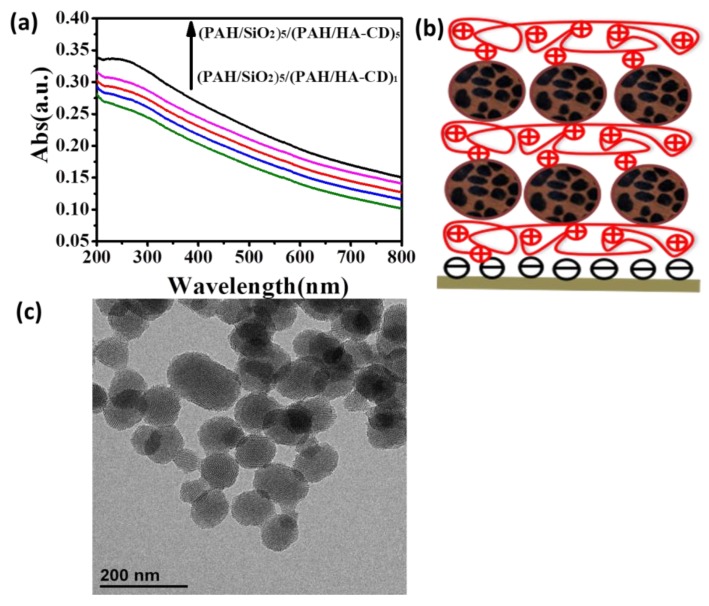
(**a**) Assembly process followed by UV-vis spectrum of (SiO_2_/PAH)_5_/(PAH/HA-CD)_5_/(PAH/DAS)_5_ multilayers; (**b**) Cartoon showing the process of layer-by-layer self-assembly; (**c**) TEM image of MSNs.

MSNs were synthetized according to the literature [49]. In brief, an aqueous solution containing of 2.0 g CTAB, 7.0 mL NaOH (2 mol L^−1^) and H_2_O (480 g) was heated for 30 min at 80 degrees with rapid stirring. To this solution, 8.31 g ethyl orthosilicate was added. The white solid appeared within two minutes. The product was further stirred at 80 degrees for two more hours, followed by centrifugation, washing with a lot of ultra-pure water, and drying in vacuum oven for 12 h. The CTAB extraction was carried at 60-degrees Celsius. This white powder was added to a mixture of ethanol (120 mL) and concentrated hydrochloric acid (1.0 mL) and stirred for 8 h at 60 degrees Celsius. The product was then centrifuged and washed with a lot of water and ethanol, followed by drying under vacuum. The diameter of meso-SiO_2_ is about 100 nm as seen in TEM images shown in Figure 1c. The surface area of the MSN is 646.715 m^2^ g^−1^ and the pore diameter of the MSN was about 3.4 nm, which was characterized via the BET instrument.

### 2.4. Layer-By-Layer Assembly Multilayer

Quartz slides cleaned by piranha solution (concentrated H_2_SO_4_/H_2_O_2_ (v:v = 7:3) ) for 10 h were immersed in PAH (aq., MW = 15,000, 1 mg ml^−1^, pH = 9) for 25 min in a typical layer-by-layer cycle. They were rinsed in ultrapure water and then dried with nitrogen. After that, the slides were dipped into the SiO_2_ solution for 25 min and rinsed and dried as before. The cycle was continued until desirable thickness was obtained and the related coating film will be designated as (PAH/SiO_2_)_n_. Generally, the LBL film was assembled in pH = 9 solution. The substrate was immersed in PAH (aq., MW = 15,000, 1 mg mL^−1^, pH = 9) for 25 min, rinsed in ultrapure water, and then dried with nitrogen. Subsequently, these slides were soaked into the HA-CD (1 mg mL^−1^) solution for 25 min, rinsed and dried as before so that (PAH/HA-CD) LBL films could be deposited onto the surface of the (PAH/SiO_2_)_n_ films. The film should be fixed on the quartz substrate to avoid bending during handling so that the good quality of multilayers on the quartz substrate can be guaranteed.

### 2.5. Peptide Loading and Release

1 mg of FITC-labeled peptide solution was dissolved in 10 mL of PBS buffer solution (pH = 7.4) in a 20 mL glass beaker at room temperature. The quartz sheets with the multilayers on the surface were then immersed in the peptide solution for 24 h. The quartz sheets were then removed and the surface was immediately rinsed with deionized water and blown dry with nitrogen. Finally, these quartz sheets were used for release experiments in PBS buffer solution.

## 3. Results and Discussions

**Scheme 1:**
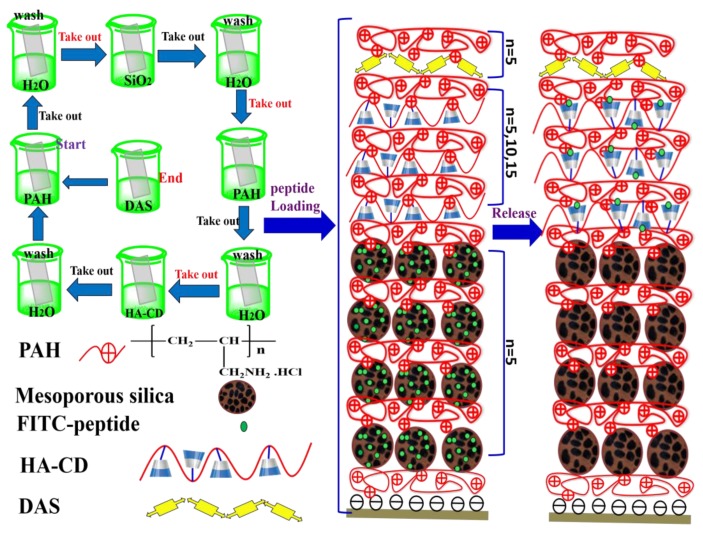
Description of the loading and release using multilayer film: polymer layers and the mesoporous silica reservoir both play an important role in fabricating functional and stable multilayer films.

**Figure 2:**
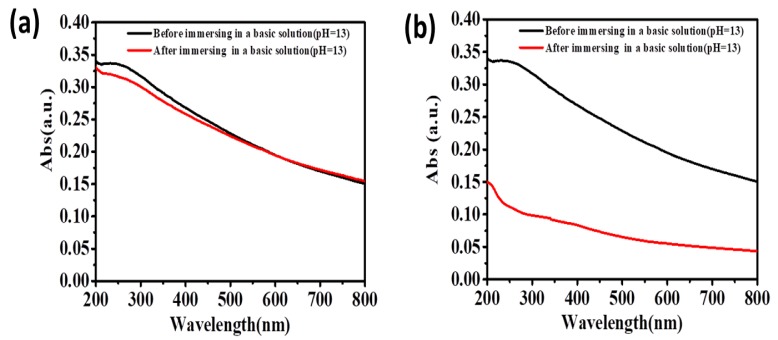
(**a**) UV-vis spectrum of the cross-linked (SiO_2_/PAH)_5_(PAH/HA-CD)_5_(PAH/DAS)_5_ film before (black) and after (red) immersion in a basic solution (pH = 13); (**b**) UV-vis spectrum of the uncross-linked (SiO_2_/PAH)_5_/(PAH/HA-CD)_5_/(PAH/DAS)_5_ film before (black) and after (red) immersion in a basic solution (pH = 13).

The surfaces of PAH and DAS are positively charged. PAH and DAS can form covalent cross-linked layers in situ under ultraviolet irradiation. The versatile multilayer films reported here were built by integrating MSN, which act as the storage space of polypeptide of FITC-peptide-Ad and the covalently cross-linking multilayers to increase the stability of the whole system of the multilayer films (Scheme 1). A multilayer film of (PAH/SiO_2_)_5_/(PAH/HA-CD)_5_ was prepared as illustrated in Figure 1a. The formation of this multilayer was monitored by UV-vis spectroscopy. The cartoon as shown in Figure 1b, displays the process of layer by layer self-assembly between MSN and PAH. The MSN used in the experiment were synthesized in our laboratory. The diameters for these MSNs vary from small to 100 nm as shown in Figure 1c. The thickness of the cross-section of multilayer films prepared on the silica substrate was about 140 nm with surface roughness of around 35 nm (Figure S2). As compared to uncross-linked (PAH/DAS)_5_ films, the stability of the (PAH/SiO_2_)_5_ multilayer films is not great and could not be enhanced, and the absorbance of the basic solution remains 32.26% of the original absorbance after the immersion as shown by the UV-vis spectra (Figure 2a,b).

UV irradiation was used to enhance the stability of the multilayer films. (PAH/DAS)_5_ multilayer films were subsequently prepared by a self-assembly method on the top of the (PAH/SiO_2_)_5_/(PAH/HA-CD)_5_ multilayers, and cross-link of (PAH/DAS)_5_ multilayer films was analyzed using UV spectra. The decrease of the absorbance peak at 340 nm indicates that the DAS decomposes and forms covalent cross-linkages within the (PAH/DAS)_5_ films. The stability of these films can be verified by soaking the cross-linked multilayer films into the basic solution. After the treatment with basic solution for 2 h, the peak absorbance of the multilayer films increased by 94.12% (Figure 2a).

The result shows that the covalently cross-linked (PAH/DAS)_5_ acts as an outermost film which contributes in the enhancement of the stability of the multilayer films within the MSNs. Based on these results, the presence of (PAH/DAS)_5_ multilayer films can enhance the stability of the uncross-linked multilayer films. It was supposed that the nano-net effect resulted in the ability of (PAH/DAS)_5_ multilayer films to improve the stability of the films by incorporating silica underneath. In addition, DAS could enter the multilayer films and also enhance the stability of the multilayer films through covalent cross-linking bonding. This effective and facile strategy can play an important role in practical purposes because of its ability in stabilizing different kinds of multilayer films.

In consideration of the above facts, a model multilayer film for the release of FITC-labeled peptides can be employed as a model drug. The host-guest interaction between CD and Ad existed on the three-dimensional scale. The cyclodextrin molecule is hydrophilic from outside and hydrophobic from inside. It indicates that the CD possesses the external hydrophilic surface and the hydrophobic central cavity.

**Figure 3:**
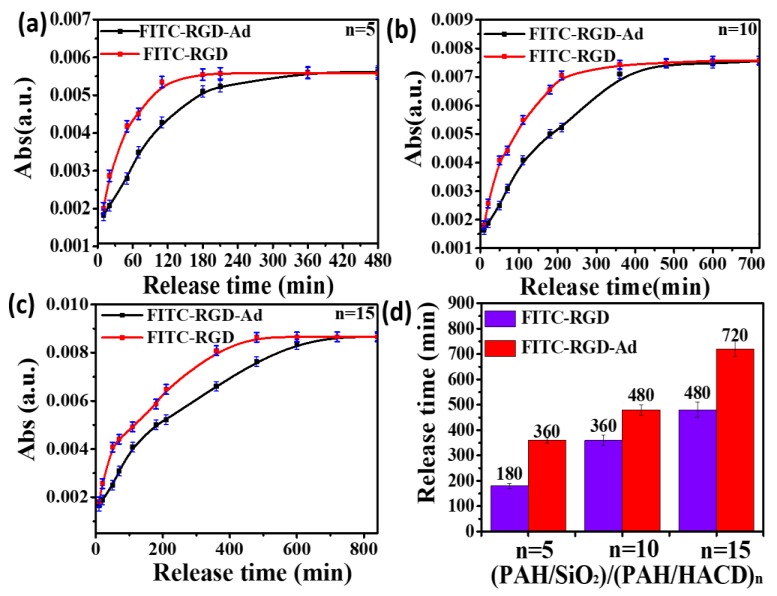
Release profiles of FITC-RGD-Ad and FITC-RGD from the cross-linked (**a**) (SiO_2_/PAH)_5_/(PAH/HA-CD)_5_/(PAH/DAS)_5_; (**b**) (SiO_2_/PAH)_5_/(PAH/HA-CD)_10_/(PAH/DAS)_5_ and (**c**) (SiO_2_/PAH)_5_/(PAH/HA-CD)_15_/(PAH/DAS)_5_ multilayer films. (**d**) the release time of FITC-RGD-Ad and FITC-RGD from different kinds of multilayer films.

**Figure 4:**
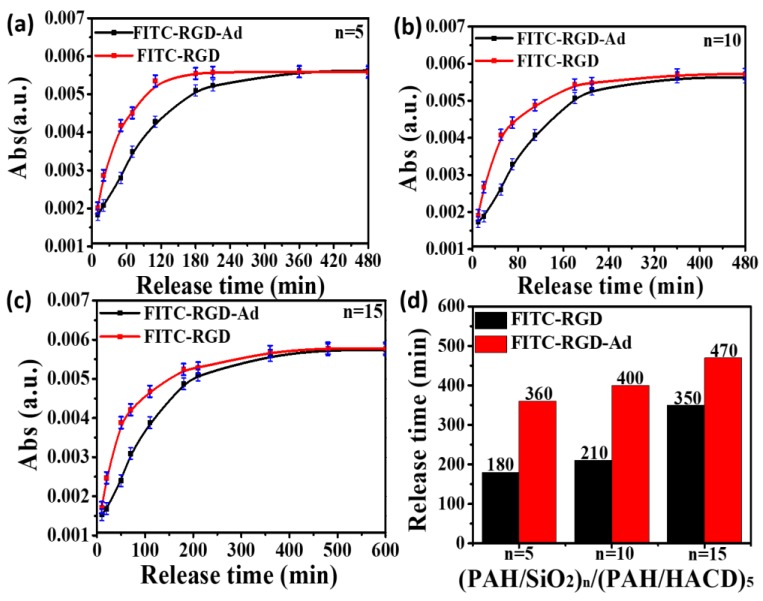
Release profiles of FITC-RGD-Ad and FITC-RGD from the cross-linked (**a**) (SiO_2_/PAH)_5_/(PAH/HA-CD)_5_/(PAH/DAS)_5_. (**b**) (SiO_2_/PAH)_10_/(PAH/HA-CD)_5_/(PAH/DAS)_5_ and (**c**) (SiO_2_/PAH)_15_/(PAH/HA-CD)_5_/(PAH/DAS)_5_ multilayer films. (**d**) The release time of FITC-RGD-Ad and FITC-RGD from different kinds of multilayer films.

Figure 3 shows the short peptides release profiles from cross-linked multilayer films. Figure 3a clearly shows that the drug release rate of FITC-RGD is faster than the drug release rate of FITC-RGD-Ad. The release curve clearly shows the release time of FITC-RGD from (SiO_2_/PAH)_5_/(PAH/HA-CD)_5_/(PAH/DAS)_5_ is about 180 min (in the red line) (Figure 3a); however, the release time of FITC-RGD-Ad from (SiO_2_/PAH)_5_/(PAH/HA-CD)_5_/(PAH/DAS)_5_ is about 360 min (in the black line) (Figure 3a). As shown in Figure 3b, the release time of FITC-RGD and FITC-RGD-Ad is about 360 min and 480 min, respectively. As shown in Figure S3, ten layers of multilayers were used for experiments to prove that the molecular size had little effect on the release rate. As shown in Figure S3, the release time of both FITC-RGD and FITC-RGD-Ad is about 480 min. As illustrated in Figure 3c, it takes about 480 min for the release of FITC-RGD, and 720 min for FITC-RGD-Ad from (SiO_2_/PAH)_5_/(PAH/HA-CD)_5_/(PAH/DAS)_5_ multilayer film. Moreover, it shows that the release profiles are very smooth in Figure 3. Despite the number of multilayers in the film being same, the release speed and release time is different. The delayed effect of the films was caused by the host-guest supramolecular interactions between cyclodextrin and adamantane and also the release process increased with number of the multilayers. In addition, the release time of different kinds of multilayer is summarized in Figure 3d. In the experiment, the controlled variable method was used to study the release of peptide. Figure 4a is the same as Figure 3a. Compared with the release profiles in Figure 3 and Figure 4a, Figure 4b clearly shows that the peptide release speed of FITC-RGD is faster than the release speed of FITC-RGD-Ad. The release time of FITC-RGD and FITC-RGD-Ad is about 210 min and 400 min, respectively. As seen in Figure 4c, the release time of FITC-RGD and FITC-RGD-Ad is 350 min and 470 min, respectively. Delayed effect of the multilayer films resulted from the host-guest supramolecular interactions, which show an increase in the release process with an increasing number of multilayers.

**Figure 5:**
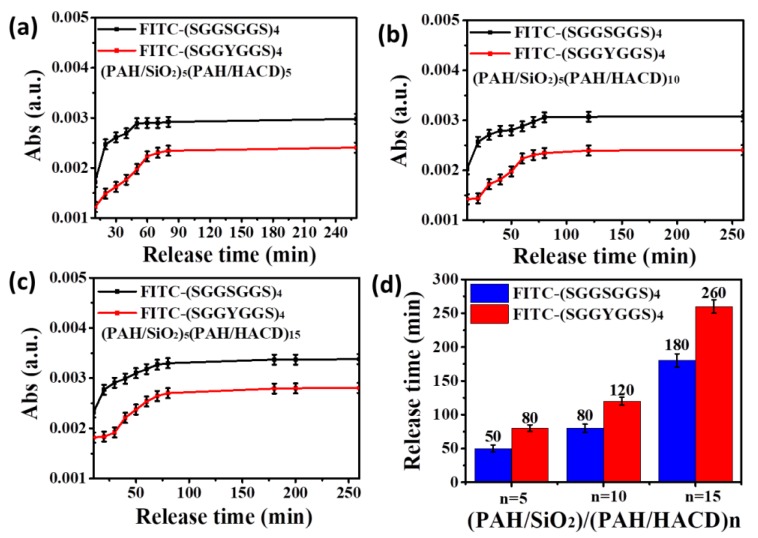
Release profiles of FITC-(SGGSGGS)_4_ or FITC-(SGGYGGS)_4_ from the cross-linked (**a**) (SiO_2_/PAH)_5_/(PAH/HA-CD)_5_/(PAH/DAS)_5_ (**b**) (SiO_2_/PAH)_5_/(PAH/HA-CD)_10_/(PAH/DAS)_5_ and (**c**) (SiO_2_/PAH)_5_/(PAH/HA-CD)_15_/(PAH/DAS)_5_ multilayer films. (**d**) The release time of FITC-RGD-Ad and FITC-RGD from different kinds of multilayer films.

It is well known that tyrosine (Y) is a hydrophobic amino acid. We infer that host-guest interactions between CD and Y will delay the release speed of Y-based peptide. For proof of concept, different kinds of multilayers were immersed into different solution of peptide, as shown in Figure 5.

As illustrated in Figure 5a, the release time of FITC-(SGGYGGS)_4_ from (SiO_2_/PAH)_5_(PAH/HA-CD)_5_(PAH/DAS)_5_ is about 50 min. This is the time taken to obtain saturated release of fluorescent probe. For FITC-(SGGSGGS)_4_ from (SiO_2_/PAH)_5_(PAH/HA-CD)_5_(PAH/DAS)_5_ this release time is 80 mins. As shown in Figure 5b, the release time of FITC-(SGGYGGS)_4_ from (SiO_2_/PAH)_5_(PAH/HA-CD)_10_(PAH/DAS)_5_ is about 80 min and around 120 min for FITC-(SGGYGGS)_4_ from (SiO_2_/PAH)_5_(PAH/HA-CD)_10_(PAH/DAS)_5_. Figure 5c shows that it takes around 180 min for the release of FITC-(SGGSGGS)_4_ and 260 min for FITC-(SGGYGGS)_4_ from (SiO_2_/PAH)_5_(PAH/HA-CD)_15_(PAH/DAS)_5_ multilayer films. The more the number of the multilayers in the film, the greater the host-guest interactions. By changing variables and adjusting parameters, it is proved that upon coming in contact with supramolecular forces, host-guest interactions between HA-CD and FITC-(SGGYGGS)_4_ delayed the release speed of fluorescent molecules as shown in Figure 5.

## 4. Conclusions

In this study, a method has been developed which can raise the stability of multilayer films made up of polymer and inorganic nanoparticles. The covalently cross-linked super stratum was formed on outermost layer of the multilayer films so that the structure of multilayers can be stabilized. The super stratum acts as a nano-net which prevents the diffusion of the nanoparticles from the substrate. It is worth mentioning that the super stratum possesses permeability for small molecules like FITC-RGD, FITC-RGD-Ad, FITC-(SGGYGGS)_4_ or FITC-(SGGSGGS)_4_. The difference in permeability of the super stratum towards nanoparticles and small molecules is utilized to synthesize a functional system for drug delivery incorporating mesoporous silica as the molecule reservoir. The construction of functional multilayer films that incorporate inorganic nanoparticles has potential promotional value benefitting from the mild synthesis conditions of the super stratum.

## Supplementary Materials

The following are available online at https://www.mdpi.com/1996-1944/12/8/1206/s1.
