# Supplementary material for: Versatile Layer-By-Layer Highly Stable Multilayer Films: Study of the Loading and Release of FITC-Labeled Short Peptide in the Drug Delivery Field
Source: Materials (Basel). 2019 Apr 12;12(8):1206. doi: 10.3390/ma12081206 (PMC6514557; doi:10.3390/ma12081206)
Supplement: Supplementary file 1 [file materials-12-01206-s001.pdf]

materials

Supplementary Materials: Versatile Layer-by-Layer Highly Stable Multilayer Films: Study of the Loading and Release of FITC-Labeled Short Peptide in the Drug Delivery Field

Kun Nie [1], Xiang Yu [1,*], Navnita Kumar [2] and Yihe Zhang [1,*]

1 Beijing Key Laboratory of Materials Utilization of Nonmetallic Minerals and Solid Wastes, National Laboratory of Mineral Materials, School of Materials Science and Technology, China University of Geosciences (Beijing), Beijing 100083, China; nk@cugb.edu.cn (K. N.)
2 Department of Chemistry and Biochemistry, University of California, Los Angeles, CA, 90095, United States; navnitakumar77@gmail.com (N. K.)
* Correspondence: yuxiang@cugb.edu.cn (X.Y.); zyh@cugb.edu.cn (Y.Z.); Tel.: +86-189-1180-7048 (X.Y.)

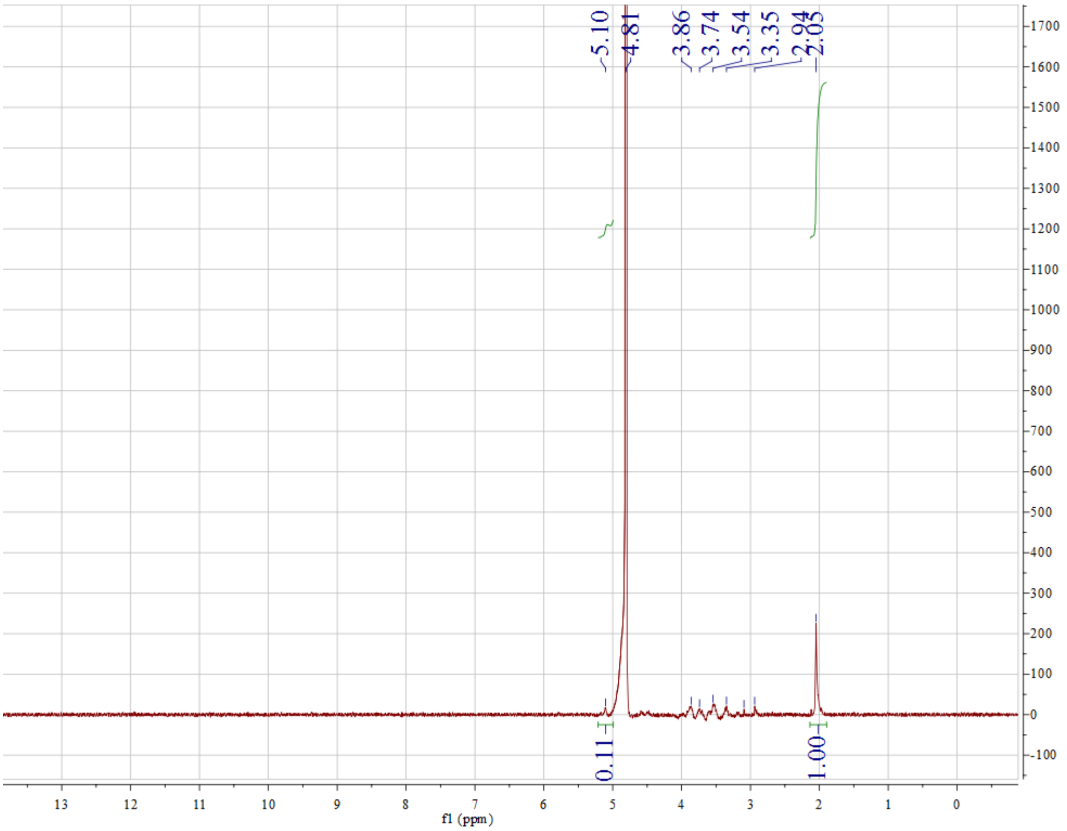

Figure S1 ^{1}H NMR spectrum (400 MHz, D_2O) of HA-CD

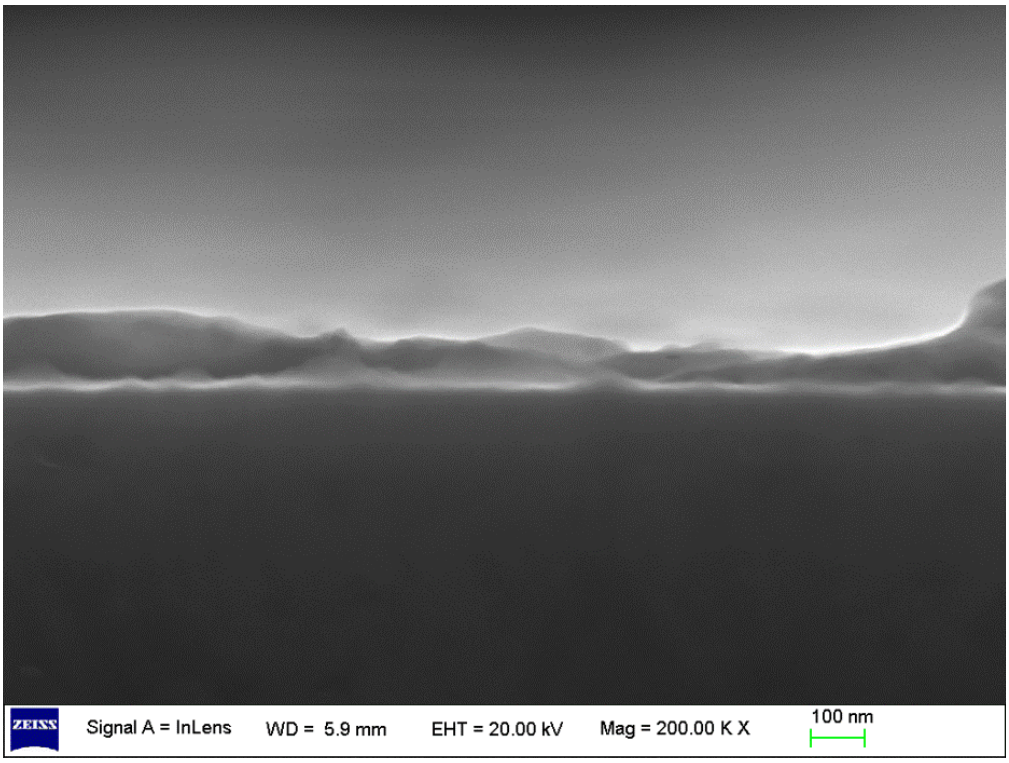

Figure S2 The SEM image of the cross-section of (PAH/SiO_2)$_5$(PAH/HA-CD)$_5$ multilayer films

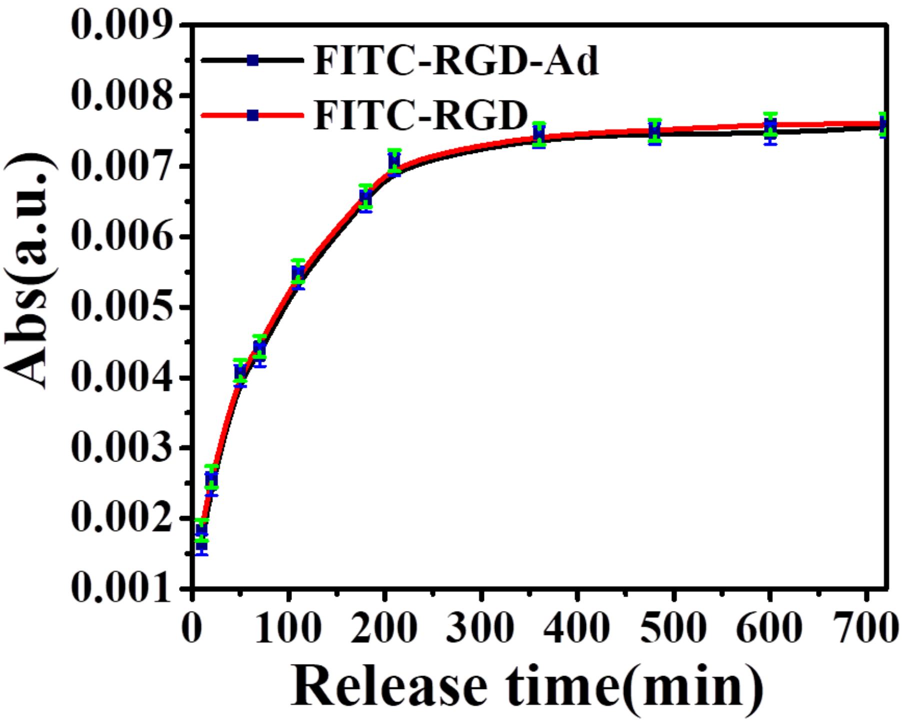

Figure S3 Release profiles of FITC-RGD-Ad and FITC-RGD from the cross-linked (SiO_2/PAH)$_5$(PAH/HA)$_{10}$(PAH/DAS)$_5$ multilayers

Table S1 The experimental ingredient for preparation of HA-CD

Product	HA(g)	MES (mL)	EDC (g)	NHS (g)	NH_2-β-CD (g)	Grafting rate (%)
**HA-CD**	0.3	40	0.285	0.342	0.1686	4.71

The table of contents (TOC)

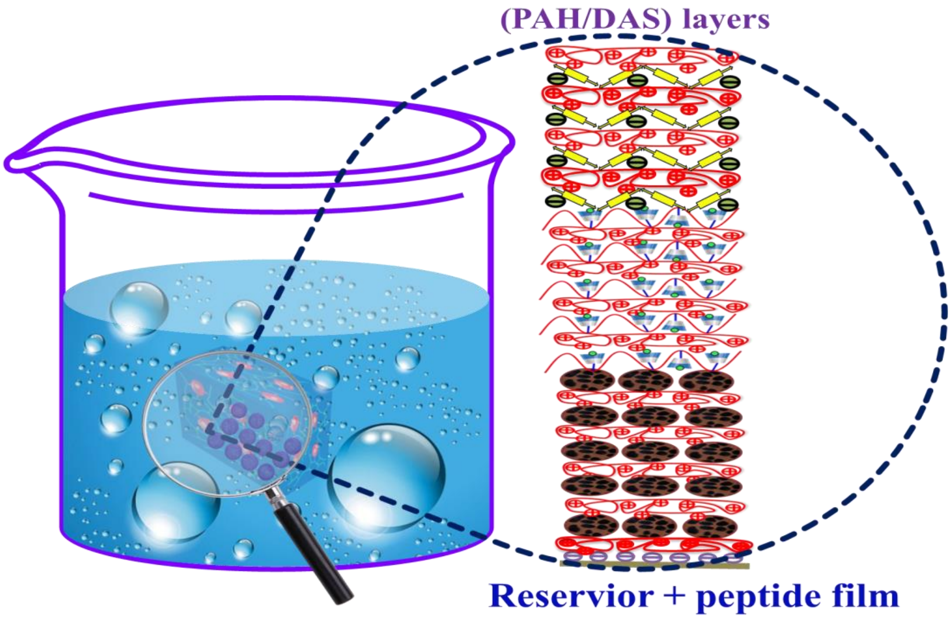

Layer-by-layer functional multilayer films that not only preserve peptides but also can adjust the magnitude of supramolecular interact
